# Quantitative Susceptibility Mapping of the Basal Ganglia and Thalamus at 9.4 Tesla

**DOI:** 10.3389/fnana.2021.725731

**Published:** 2021-09-16

**Authors:** Vinod Jangir Kumar, Klaus Scheffler, Gisela E. Hagberg, Wolfgang Grodd

**Affiliations:** ^1^Max Planck Institute for Biological Cybernetics, Tübingen, Germany; ^2^Biomedical Magnetic Resonance, University Hospital and Eberhard-Karl’s University, Tübingen, Germany

**Keywords:** QSM, thalamus, basal ganglia, high field MRI, myelin, iron

## Abstract

The thalamus (Th) and basal ganglia (BG) are central subcortical connectivity hubs of the human brain, whose functional anatomy is still under intense investigation. Nevertheless, both substructures contain a robust and reproducible functional anatomy. The quantitative susceptibility mapping (QSM) at ultra-high field may facilitate an improved characterization of the underlying functional anatomy *in vivo*. We acquired high-resolution QSM data at 9.4 Tesla in 21 subjects, and analyzed the thalamic and BG by using a prior defined functional parcellation. We found a more substantial contribution of paramagnetic susceptibility sources such as iron in the pallidum in contrast to the caudate, putamen, and Th in descending order. The diamagnetic susceptibility sources such as myelin and calcium revealed significant contributions in the Th parcels compared with the BG. This study presents a detailed nuclei-specific delineation of QSM-provided diamagnetic and paramagnetic susceptibility sources pronounced in the BG and the Th. We also found a reasonable interindividual variability as well as slight hemispheric differences. The results presented here contribute to the microstructural knowledge of the Th and the BG. In specific, the study illustrates QSM values (myelin, calcium, and iron) in functionally similar subregions of the Th and the BG.

## Introduction

Thalamus (Th) and basal ganglia (BG) are the major subcortical structures within the human brain housing a variety of cerebral functions. Here, Th serves as the central control and integration center. It is also referred to as the “gateway to the cortex” (Jones, 2007; Sherman and Guillery, 2009). The term BG defines a group of closely connected cell masses situated at the base of the telencephalon and on the top of the mesencephalon surrounding the adjacent diencephalon. The BG is involved in selective behavior, motor learning, and the control of dopamine neuron activity and value-based decisions (Kelly and Strick, 2004). The BG circuits in rodents and primates had most likely evolved already at the dawn of vertebrate evolution (Nieuwenhuys et al., 2008). They classically refer to three large subcortical nuclear masses. The caudate nucleus (NC), putamen (PU), and pallidal complex are composed of external (GPe) and internal segments (GPi) of globus pallidus (GP) and ventral pallidum. In addition, two closely related structures, substantia nigra and subthalamic nucleus are generally included as components of BG (Lanciego, 2012), but they will not be considered here. One part of BG encompassing NC and PU is the penetration by fascicles of the cortico- and striatofugal axons, also assigned as the striatum. The striatum (NC + GP) hosts the largest subcortical cell mass of the brain; it is functionally divided into a ventral and a dorsal part.

Due to the importance of Th and BG in health and disease (Herrero et al., 2002; DeLong and Wichmann, 2007; Paprocka et al., 2020), several *in vivo* studies have been performed by using different MRI modalities to investigate their functional and anatomical properties (Wu et al., 2012; Imai et al., 2018; Filyushkina et al., 2019). In particular, quantitative susceptibility mapping (QSM) provides a novel MRI contrast mechanism to quantify iron and biomarkers, including myelin, calcium, gadolinium, and super-paramagnetic iron oxide nanoparticles (Liu et al., 2015; Möller et al., 2019). The QSM provides excellent subcortical gray-matter nuclei contrast compared to the conventional MRI sequences such as T1- and T2-weighted. Therefore, QSM has been repeatedly used to depict typical subcortical structures and pathological alterations in Parkinson’s, Alzheimer’s, and other such diseases (Wang and Liu, 2015; Santin et al., 2016; Shahmaei et al., 2019; Liu et al., 2021). However, previous QSM work has also revealed that a refined depiction of subcortical anatomy requires field strength > 3 Tesla to sufficiently assess the finer details of subcortical structures (Loureiro et al., 2018; Alkemade et al., 2020).

As the Th and BG are composed of cell bodies with dendritic arborizations and densely myelinated connectivity hubs containing both projecting and receiving fibers, we hypothesize that in the QSM map, Th and BG show diamagnetic and paramagnetic sources of variable composition. Few studies depict that the microstructural properties of Th and BG using up to 7T MR QSM maps. Given the significant functional and structural importance of Th–BG, there is a motive to investigate it in a finer scale of resolution at higher field strengths. We, therefore, investigated diamagnetic and paramagnetic sources by using a predefined functional parcellation of Th and BG in a sample of 21 normal subjects by using QSM with ultrahigh-resolution data obtained at 9.4 Tesla.

## Materials and Methods

### Subjects and MRI Acquisition

Twenty-one healthy volunteers (thirteen male, eight female, 20–56 years old) without any neurological disorders were screened through a qualified rigorous safety assessment of a qualified doctor, and scanned at 9.4 Tesla (Siemens Medical Solutions, Erlangen, Germany) by using a 16-channel transmit/31-channel receive array (Shajan et al., 2014). B1-mapping, anatomical MP2RAGE images (Hagberg et al., 2017) and monopolar multiecho 3D gradient echo (GRE) images with 5 echoes and echo times, TE = 6–30 ms in steps of 6 ms; a repetition time TR = 35 ms; and nominal FA = 11° with a voxel size of 375 × 375 × 800 μm were acquired with an axial prescription.

### Data Analysis

**FIGURE 1:**
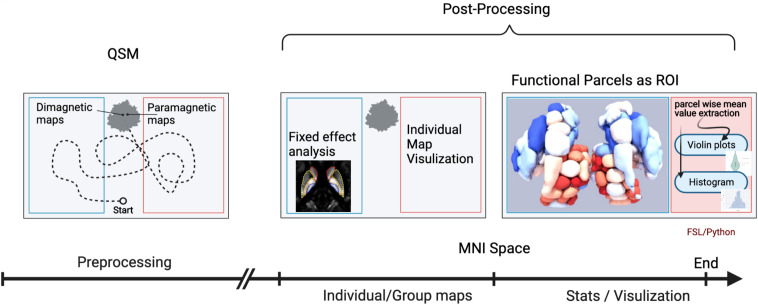
Workflow of the quantitative susceptibility mapping (QSM) evaluation. An illustration of the workflow depicts the analysis steps. The first analysis step was a mix of several preprocessing steps: (1) generation of QSM maps by using background removal imposing image smoothness and moderate contrast; (2) transformation into standard MNI brain space, after co-registration to anatomy data of each individual subject with known spatial transformation (DARTEL), and reslicing onto a grid with isotropic 400 μm voxels using trilinear interpolation; (3) bifurcation/subdivision into diamagnetic and paramagnetic components. In the next step, the diamagnetic and paramagnetic maps were bifurcated from the normalized QSM maps. Afterward, in the post-processing steps, individual as well as group fixed maps were visualized. The last analysis step encompasses statistics and visualization of the group results. The graphical workflow was created with BioRender.com https://app.biorender.com/biorender-templates.

A brief overview of the analysis workflow is illustrated in Figure 1.

### Preprocessing

Quantitative susceptibility mapping maps were reconstructed and preprocessed by using coil offset correction and adaptive coil combination before a Laplacian unwrapping, phase-based masking, as described previously (Hagberg et al., 2020). Whole-slab phase referencing followed by the variable-kernel (VSHARP) background removal and dipole inversion was done by using STI-studio. After coregistration to anatomical images, the QSM maps were normalized to the MNI space and multiplied by 1,000 to obtain standardized data in the ppb range.

### Postprocessing

In the first step, the MNI-spaced atlas were resliced to 400-micron native data resolution by using FMRIB Software Library (FSL) (Jenkinson et al., 2012). The choice to bring native space data into a common brain space compromises between obtaining sufficient anatomical detail and minimizing distortions due to the nonlinear transformation. Positive QSM values were assigned to paramagnetic maps, setting all other voxels to zero (>0) and vice versa; negative values (<0) were assigned to the diamagnetic maps.

### Fixed Effect and Individual Maps

**FIGURE 2:**
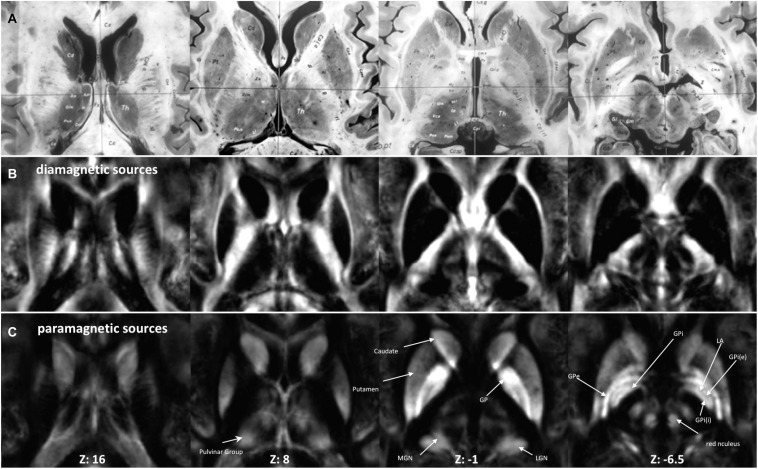
Anatomy and MRI of thalamus (Th) and basal ganglia (BG): **(A)** four selected slices were adapted (Schaltenbrand and Bailey, 1959) **(B,C)** Corresponding QSM images of diamagnetic and paramagnetic sources. GPe, external segments of the globus pallidus; GPi, internal segments of the globus pallidus; GPi (e), subsegment of internal segments of the globus pallidus; GPi (i), subsegment of internal segments of the globus pallidus; LGN, lateral geniculate nucleus; MGN, medial geniculate nucleus; LA, lamina accessoria.

**FIGURE 3:**
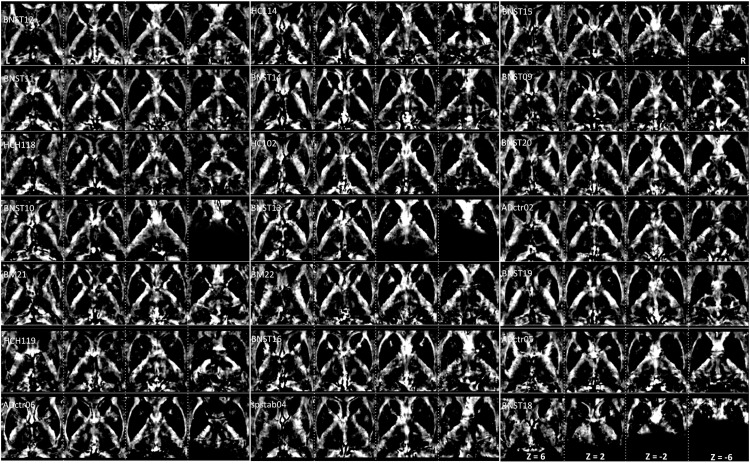
Interindividual maps: QSM images of diamagnetic sources (myelin and calcium, etc.) were obtained from 21 subjects ordered with increasing age. The grayscale color code indicates brighter values in the highly myelinated structures. In the grayscale, brighter values indicate higher and darker lower values. Note the highest values in the internal capsule: Th, NC, PU, and GP display lower values. However, in the quantitative analysis, the difference converges, as the scale of differences in the visualization is heavily biased by the internal capsule fiber contrast. Three subjects (BNST10, BNST13, and BNST18) show only partial coverage.

The fixed effect (Figure 2) and individual maps were visualized for diamagnetic and paramagnetic contributions within BG and Th (Figures 3, 5).

**FIGURE 4:**
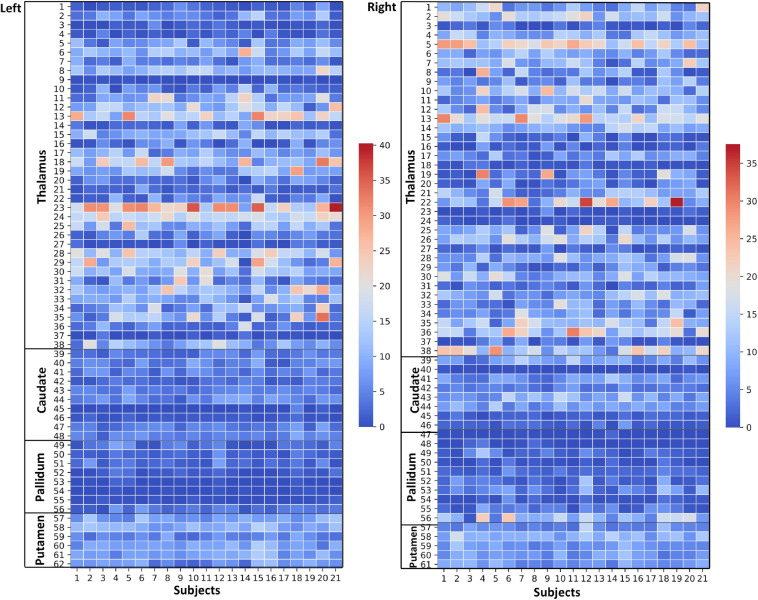
Thalamus and Basal Ganglia (Diamagnetic sources, i.e., Myelin, Calcium, etc.): *Left hemisphere:* Mean diamagnetic source values for all parcels in all subjects in the left hemisphere. Each column represents each subject. Each row represents a functional parcel. The total number of functional parcels is 62, i.e., Thalamus (38) + Caudate (10) + Pallidum (8) + Putamen. (6) *Right hemisphere:* Mean diamagnetic source values for all parcels in all subjects in the right hemisphere. The total number of ICP functional parcels is 61 i.e., Thalamus (38) + Caudate (8) + Pallidum (10) + Putamen (5). Note the higher values in some thalamus parcels compared to other structures.

**FIGURE 5:**
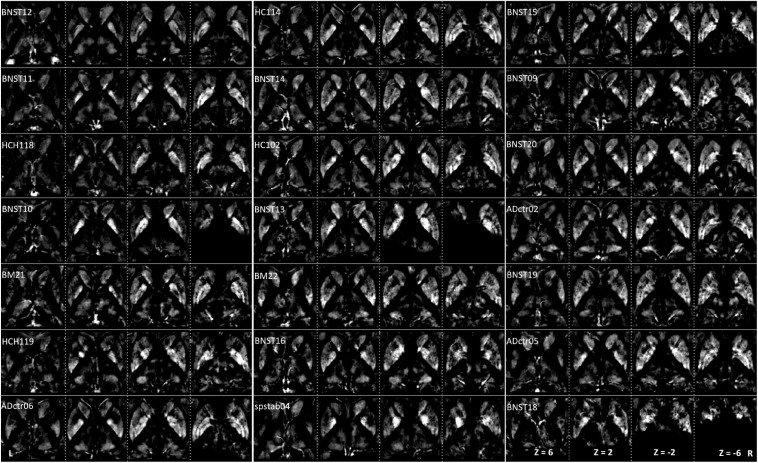
Intervididual maps: QSM images of paramagnetic sources (iron, etc.) obtained from 21 subjects ordered with increasing age. The grayscale color code indicates brighter values in the highly myelinated structures. Note the highest values in the pallidum, caudate, at the specific sites in putamen and thalamus. Darker values indicate lower values. Three subjects (BNST10, BNST13, BNST18) show only partial coverage.

### Parcel-Specific Analysis

**FIGURE 6:**
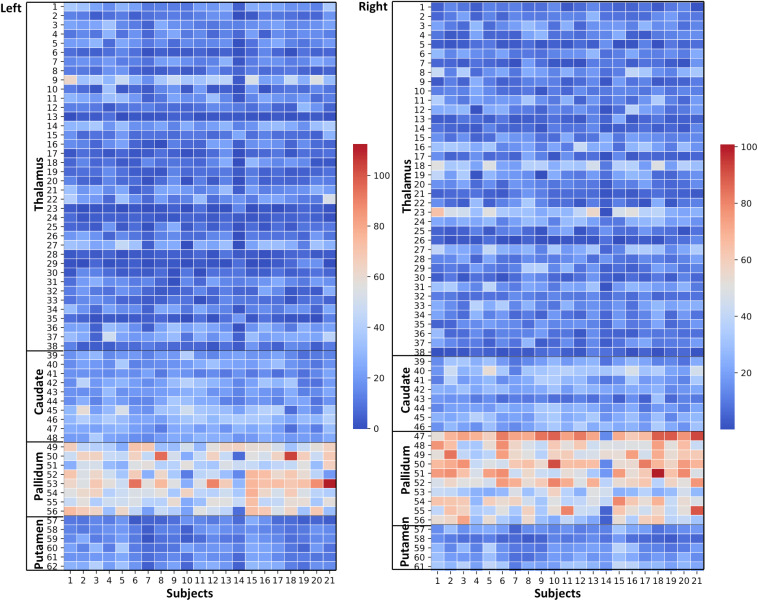
Effects of paramagnetic sources (iron) in Th and BG: *Left hemisphere*: Mean diamagnetic source values for all parcels in all subjects. Each column represents each subject. Each row represents a functional parcel. The total number of ICP functional parcels are 62, i.e., Th (38) + caudate (10) + pallidum (8) + putamen (6). Note the higher mean values in pallidum and some of Th parcels compared to other structures. *Right hemisphere*: Mean diamagnetic source values for all parcels in all subjects. Each column represents each subject. Each row represents a functional parcel. The total number of ICP functional parcels are 61, i.e., Th (38) + caudate (8) + pallidum (10) + putamen (5). Note the higher mean values in pallidum and some of Th parcels compared to other structures.

**FIGURE 7:**
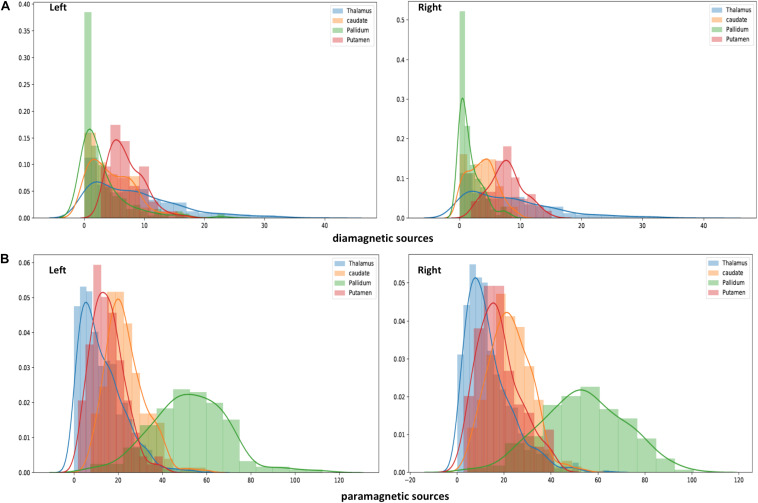
Histogram of QSM-values in diamagnetic **(A)** and paramagnetic sources **(B)**: Distribution of mean di- and paramagnetic sources within Th and BG. The different colors indicate corresponding structures. The X-axis depicts different QSM values and the Y-axis depicts the amount of each value. Note the differences, where the diamagnetic source, the pallidum, shows the first peak, caudate the second, putamen the third, and Th in the last, while for the mean paramagnetic source, Th shows the first peak, putamen the second, caudate the third, and pallidum the last. Overall, the diamagnetic sources span a more narrow range of values than paramagnetic sources.

**FIGURE 8:**
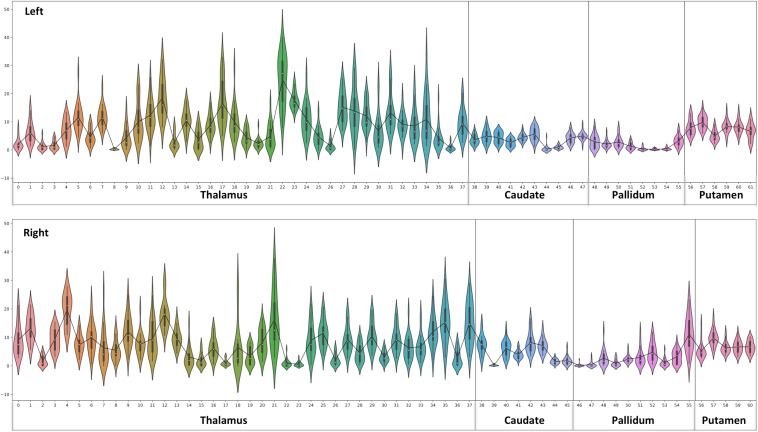
Violin plot of Diamagnetic sources, i.e., myelin and calcium: The violin plot highlights only the mean of each parcel. The black-line plot shows only the mean values of all parcels in all subjects. First, note the overall lower values for the caudate and pallidum, and in contrast, putamen and several parcels in the Th show consistently higher pallidum parcel values. Second, the violin plot reveals inter-individual variability.

A parcel-specific analysis was performed by using an existing functional parcellation of Th, NC, PU, and GP. The robust and reproducible parcellation of Th and BG was achieved by using instantaneous correlation analysis (Kumar et al., 2017; van Oort et al., 2018) at 7 Tesla HCP rfMRI data (Kumar et al., 2019). In the next step, nuclei-specific QSM means were computed for the left and right hemispheres in the diamagnetic and paramagnetic maps. Subject-specific mean values (Figures 4, 6), histogram (Figure 7), and violin plots (Figures 8, 10) were visualized to depict respective comparisons, i.e., quantitative overview and descriptive statistics.

**FIGURE 9:**
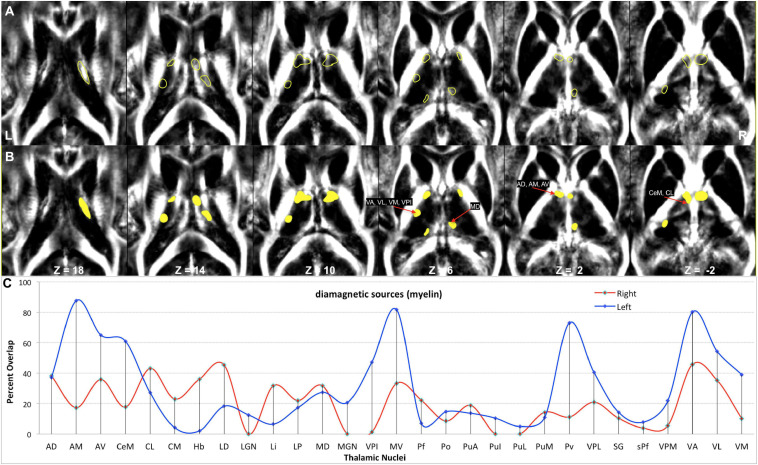
Depiction of values (>10 ppb) of diamagnetic sources in Th. **(A)** The spatial location of diamagnetic parcels with mean values > 10 ppb within the Th. BG does not reveal areas exceeding a threshold of >10 ppb. The yellow color outline encircles thalamic parcels. **(B)** Colored version of the first row illustrates regions with higher values. **(C)** Calculated overlap map of diamagnetic parcels with the thalamic nuclei according to the atlas of Morel show higher values in the anterior Th (AD, AM, and AV), intralaminar nuclei (CeM and CL), the motor and sensory nuclei (VA, VL, VM, VPI, and MV), and in Pv as well as the MD nuclei.

**FIGURE 10:**
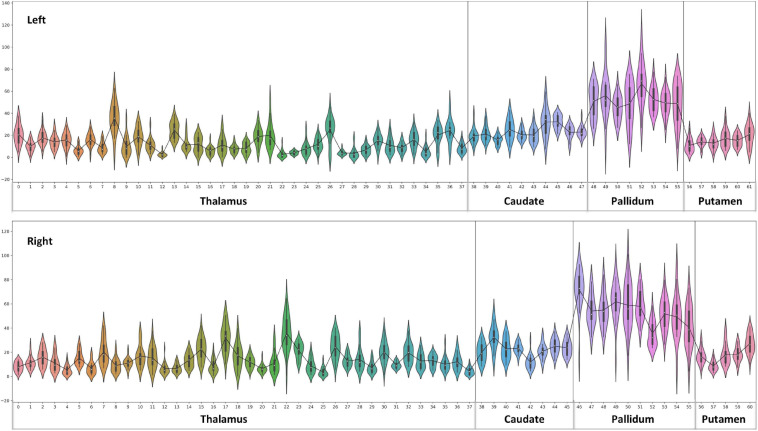
Violin plot of paramagnetic sources (iron): the violin plot highlights only the mean of each parcel. The black line plot shows only the mean values of all parcels in all subjects. First, note the overall lower values for the Th, caudate, and putamen contrast to consistent higher pallidum parcels values. Second, the violin plot reveals inter-individual variability.

### Anatomical Assignments

**FIGURE 11:**
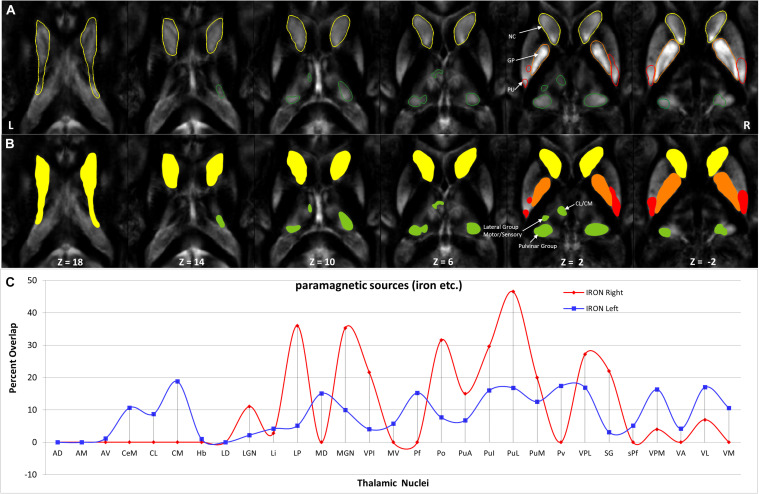
Depiction of value (>20) of paramagnetic sources in Th and BG: **(A)** Outline depiction of the paramagnetic parcel with mean values > 20 for the caudate (yellow), putamen (red), pallidum (orange), and Th (green). Note that the striatum (pallidum and caudate) encircle most of their structure above the threshold. **(B)** Colored version of the first row illustrates higher value regions. **(C)** Calculated overlap with the Thatlas shows higher values in the nuclei of the posterior Th (Po, PuA, Pul, and PuL), the relay nuclei (MGN, VPI, VPL, VPM, and VL), and the high-order intralaminar nuclei (CL and CM).

Hotspots with higher mean values (Figures 9, 11) in the diamagnetic (>10) and paramagnetic (>20) maps were analyzed for their anatomical assignments within Th by using the atlas of Morel (Krauth et al., 2010).

## Results

### Group Fixed Effect and Individual Maps

The group fixed effect maps exhibited an overview of significant paramagnetic and diamagnetic sources within Th and BG (Figure 2), and depicted the complex anatomical landscape of Th, NC, PU as well as of GPe and GPi of GP (Figure 2). The GPi of the GP further possesses two subsegments separated by lamina accessoria. Surprisingly, the anatomical observations indicate that not all individuals contain both subsegments within the GPi of the pallidum. The group fixed effect and the individual maps (between 60 and 70%) both possess subsegments of the internal segment of the pallidum (Figure 5). The individual-specific visualization of the di/paramagnetic maps depicts gross-level similarities and slight differences (Figures 3–6). The similarities comprise Th, NC, PU, and GP. Differences include variations in intensity and shape (i.e., contours, elongation, and curl). The reasons for these underlying individual variability variations might be due to technical and methodological reasons (shimming and SNR, etc.) and individual anatomical variations, including age and sex. However, details have to be addressed in future studies and when a larger data size becomes available.

### Diamagnetic Sources (Myelin and Calcium, etc.) Mapping of BG and Th

The general patterns remain with lower diamagnetic values in BG in comparison with Th. Several parcels exhibit lower values in Th, and only a few parcels show higher diamagnetic values (Figures 4, 8). The violin plot of all mean values for all the subjects revealed a variable pattern in Th and BG with lower and others with higher myelin values. Therefore, we outlined only parcels showing higher values within Th and BG (Figures 4, 8). In differentiating BG parcels, NC and GP show similar values but PU exhibits slightly higher values (Figure 8). A combined histogram of Th and BG revealed an exciting distribution (Figure 7A) as GP shows the lowest values followed by NC and PU. The variation within Th was smoothened and depicted in a separate histogram (Figure 7A).

### Paramagnetic Sources (Iron, etc.) Mapping of BG and Th

Paramagnetic (iron, etc.) mapping of the left and right BG and Th revealed higher values in GP in contrast to NC, PU, and Th (Figures 6, 10). The histogram of all mean values from all the subjects, i.e., violin plots, revealed a slightly variable pattern within Th with a somewhat higher pattern in NC and PU. The GP shows the highest values compared with Th, NC, and PU (Figure 10). Intuitively, the histogram depicts a gradient organization (Figure 7B). The first peak corresponds to Th, afterward to PU and NC, and the highest values correspond to GP (Figure 7B). A similar observation remains consistent in the violin maps also (Figure 10). The detailed, subject-level overview also depicts substantial interindividual and laterality differences (Figure 6). However, we did not delve further into the interindividual and lateral variability analysis due to limited individual data.

### Anatomical Assignments of the Higher Values Within Th

The higher value, which surpassed the mean value analysis of diamagnetic sources by more than >10 ppb and paramagnetic by more than 20 ppm, revealed a characteristic spatial distribution comprising a selected set of parcels within Th (Figures 9, 11).

In comparing those parcels with the thalamic nuclei as assigned in the atlas of Morel (Krauth et al., 2010), we found that the core (MGN, VPI, VPL, VPM, and VL), the matrix (intralaminar nuclei: CL and CM), and the pulvinar nuclei (PuA, Pul, and PuL) exhibit higher diamagnetic sources, i.e., myelin, calcium, etc. (Figure 9). In contrast, paramagnetic sources were partly dominant in the pulvinar (PuA, Pul, PuL, and PuM), the anterior (AD, AM, and AV), the intralaminar (CeM and CL), the lateral (VA, VL, VM, VPI, and MV), and the medial group (MD) (Figure 11). Interestingly, there was an agreeable laterality difference in the overlap.

## Discussion

### QSM and Functional Anatomy of Th and BG

Experimental findings in rodents and nonhuman primates have shown that the BG receives and processes cortical inputs and returns them via the midbrain and Th to the cortex. Based on these experiments, a series of functionally segregated and parallel-connected basal Th-frontal loops are postulated (Mink, 2013), which provide functionally relevant information from different frontal cortical areas for further processing. These projections to the striatum have a roughly topographical organization, in which the somatosensory and motor cortex project to the posterior PU and the prefrontal cortex to the anterior caudate. It has been suggested that the topographic relationship between the cerebral cortex and the striatum provides a basis for the segregation of functionally different circuits in the BG (Kelly and Strick, 2004; Obeso et al., 2008; Rodriguez-Oroz et al., 2009). Therefore, our study investigates diamagnetic and paramagnetic sources in the functional parcels within Th and BG by using 9.4 T QSM data to assess possible components of such segregated and parallelly connected basal Th-frontal loops.

In our awareness, such detailed parcel-specific analysis has not been performed earlier for Th, NC, PU, and GP. The study relies on the large sample of 7 Tesla data from the HCP project (Van Essen et al., 2012) used for a sample-driven rfMRI-based parcellation (Kumar et al., 2019). The used parcellations employed an instantaneous correlation analysis (Kumar et al., 2017; van Oort et al., 2018) to determine a stable and reproducible estimation of functional anatomy. However, an important future scope is to investigate Th and BG using different functional anatomy atlases. Such work could enhance our understanding by delivering more microstructural details of the underlying anatomy. The main idea of such a functional anatomical investigation should be to find spatio-temporal similarities in the structural space. Achieving such a functional, more-detailed anatomical map *in vivo* will expose us to a variety of novel questions, e.g., How does the QSM render on the functional space? Therefore, we retained a preliminary analysis. The major work remains to comprehend the relationship between QSM measures and functional anatomy.

### Ultrahigh Field and Th and BG

The more insufficient spatial resolution at lower field strengths poses several perceptual limitations concerning detailed visualization of Th and BG (Forstmann et al., 2017). First, because of the inadequate precision to depict refined anatomical structures, i.e., “Where are we? What do we see?” Therefore, higher field strength like here at 9.4 T provides a much higher signal-to-noise ratio than standard 3 T (3.10 ± 0.20) and 1.76 ± 0.13 from 7 to 9.4 T (Pohmann et al., 2016), yielding superior data quality. However, given the anatomical complexity of Th and BG, much more research is warranted concerning MR-physics, sequences, artifact removal, and issues at higher field strengths.

Due to the unavailability of rsfMRI at 9.4 Tesla in our subjects, we used functional parcellation masks from the 7 T HCP data set, which contains a large sample of rsfMRI data, allowing a reliable and reproducible functional parcellation of the Th and BG.

### Interindividual Variability

There is interindividual variability, intraparcel differences, and slight differences between the hemispheres. It is well-established that the brain varies concerning interindividual, laterality, gender, and age. However, how such variability reflects properties of Th and BG in the microstructural space remains to be investigated. The reported variabilities could be due to methodological issues and individual properties such as gender and age. Thus, a larger sample of data is needed to sufficiently model the individual variability.

### Diamagnetic and Paramagnetic Sources in Th and BG

We found a higher contribution of paramagnetic sources in the GP parcels in contrast to the NC, PU, and Th parcels in descending order. The diamagnetic sources revealed substantial contribution in BG compared with Th because GP contains large dendritic arborizations packaged parallel to one another as the three-dimensional shape of flat disks (Yelnik et al., 1984). Among other reasons, such structural differences might be the reason why the pallidum shows relatively higher iron than Th, NC, and PU (Perng et al., 2021).

The diamagnetic sources are higher in Th compared with NC, PU, and GP. The Th encompasses distinct calcium-containing nuclei (Jones, 1998) as all nuclei exhibit a variable composition of parvalbumin and calbindin. The parvalbumin is a small, stable calcium-binding protein, and calbindin contains four active calcium-binding domains. In addition, Th contains myelinated fibers and lamina. Therefore, we observed higher values within Th. Several nuclei of Th receive sensory inputs and motor outputs and are densely connected via the axons; these are especially true for nuclei such as the MGN, VPI, VPL, VPM, and VL, in which we correspondingly observed higher diamagnetic values. The intralaminar nuclei are located within fibrous white matter bundles and show higher myelin values, i.e., CL and CM parcel space. The higher motor and sensory nuclei values (Figure 9) align with the higher myelin values in the connected motor and sensory cortex (Glasser and Van Essen, 2011). The intralaminar nuclei facilitate rapid communication between the brainstem and the cortex. The pulvinar group (PuA, Pul, and PuL) receives input from the structures such as the superior colliculus and densely communicates with the cortex. It also shows the higher diamagnetic values.

## Conclusion

The study reveals a detailed functionally defined parcel-specific delineation of diamagnetic and paramagnetic sources in BG and Th. We found a more substantial contribution of paramagnetic sources in the pallidum in contrast to the caudate, PU, and Th in descending order. The diamagnetic sources revealed considerable contribution in BG compared with Th. In addition, our study shows a detailed anatomy-specific existence of diamagnetic and paramagnetic sources in BG and Th. However, interindividual variability and hemispheric differences of paramagnetic and diamagnetic sources were found in our group.

The anatomical assignments of the hotspot of diamagnetic and paramagnetic sources within Th revealed an association with a number of different nuclei. The parcels were located within core nuclei (MGN, VPI, VPL, VPM, and VL) and the matrix nuclei (intralaminar nuclei: CL, CM, and pulvinar: PuA, Pul, and PuL) exhibited higher diamagnetic sources, i.e., myelin and calcium. In contrast, paramagnetic sources were dominant in the pulvinar (PuA, Pul, PuL, and PuM), anterior (AD, AM, and AV), intralaminar (CeM and CL), and lateral nuclei group (VA, VL, VM, VPI, and MV) as well as in Pv and MD.

However, further future work is needed to comprehend the relationship between QSM maps and functional anatomy in general. Furthermore, a larger sample of data is required to examine age and gender effects and improve our understanding of the microstructures of Th and BG.

## Data Availability Statement

The data analyzed in this study is subject to the following licenses/restrictions: The second last authors of the manuscript acquired the dataset. Requests to access these datasets should be directed to gisela.hagberg@tuebingen.mpg.de.

## Ethics Statement

The participants provided their written informed consent to participate in this study. The studies involving human participants were reviewed and approved by Ethics Committee of University of Tuebingen.

## Author Contributions

VK performed the analysis, prepared figures, and wrote the manuscript. KS contributed to the QSM data acquisition, feedback, and editing of the manuscript. GH contributed to the high-quality QSM data acquisition, preprocessing the data, editing the manuscript, and valuable feedback. WG contributed immensely in feedback, wrote the introduction/manuscript, and helped figure preparation. All authors contributed to the article and approved the submitted version.

## Conflict of Interest

The authors declare that the research was conducted in the absence of any commercial or financial relationships that could be construed as a potential conflict of interest.

## Publisher’s Note

All claims expressed in this article are solely those of the authors and do not necessarily represent those of their affiliated organizations, or those of the publisher, the editors and the reviewers. Any product that may be evaluated in this article, or claim that may be made by its manufacturer, is not guaranteed or endorsed by the publisher.

## Abbreviations

Th: thalamus
BG: basal ganglia
QSM: quantitative susceptibility mapping
NC: caudate nucleus
PU: putamen
GP: globus pallidus
GPe: external segments of the globus pallidus
GPi: internal segments of the globus pallidus
T: Tesla.
